# HOLD-B: A Practical Score for Early Risk Stratification in Upper Gastrointestinal Bleeding

**DOI:** 10.3390/medicina62081476

**Published:** 2026-07-30

**Authors:** Ecem Ermete Güler, Ejder Saylav Bora, Efe Kanter, Nazlı Özçelik, Elif Eryurt Öz, Mustafa Agah Tekindal

**Affiliations:** 1 Department of Emergency Medicine, Izmir Ataturk Training and Researcing Hospital, 35360 Izmir, Turkey; 2 Department of Emergency Medicine, Izmir Katip Celebi University Faculty of Medicine, 35360 Izmir, Turkey; saylavbora@hotmail.com (E.S.B.); efekanter@hotmail.com (E.K.); nazliozcelik9922@gmail.com (N.Ö.); elif_eryurt@hotmail.com (E.E.Ö.); 3 Department of Biostatistics, Izmir Katip Celebi University Faculty of Medicine, 35360 Izmir, Turkey; matekindal@gmail.com

**Keywords:** upper gastrointestinal bleeding, emergency department, risk stratification, hemoglobin, lactate, blood urea nitrogen, diastolic blood pressure, prognostic score

## Abstract

*Background/Objective*: Early identification of high-risk patients with acute upper gastrointestinal bleeding (UGIB) is essential for appropriate triage, timely intervention, and optimal resource allocation in the emergency department (ED). Existing risk scores have limitations in practicality and predictive performance. We aimed to develop and internally validate a novel pre-endoscopic score based solely on readily available ED variables to predict clinically significant high-risk outcomes in UGIB. To develop a novel pre-endoscopic risk score using routinely available emergency department parameters and to evaluate its predictive performance for clinically significant high-risk outcomes in patients with acute upper gastrointestinal bleeding. *Methods*: This retrospective single-center prognostic model development study with internal validation included 312 adults with endoscopy-confirmed non-variceal UGIB presenting to a tertiary ED between January 2023 and January 2025. High-risk status was defined as the occurrence of at least one of the following during hospitalization: blood transfusion, endoscopic hemostatic intervention, intensive care unit admission, rebleeding within 30 days, or in-hospital mortality. This composite endpoint included both clinical outcomes and management-related interventions. Independent predictors were identified using multivariable logistic regression. The HOLD-B score (Hemoglobin, Lactate, Diastolic blood pressure, and Blood urea nitrogen) was derived from significant variables. Discriminative performance was evaluated using receiver operating characteristic (ROC) analysis and compared with established scores. *Results:* Of the study population, 51.6% were classified as high risk. Hemoglobin ≤8.05 g/dL, blood urea nitrogen >44.5 mg/dL, lactate >2.15 mmol/L, and diastolic blood pressure ≤67.5 mmHg were independent predictors of high-risk status. The HOLD-B score (0–10 points) demonstrated good discriminative performance (AUC 0.798; 95% CI 0.749–0.846), outperforming Rockall (0.687), GBS (0.733), AIMS65 (0.576), ABC (0.626), ABL (0.716), and Pre-RS (0.599). At a cutoff ≥4, the HOLD-B score yielded 71.4% sensitivity and 77.5% specificity. However, in a sensitivity analysis excluding blood transfusion from the composite endpoint, the AUC decreased from 0.798 to 0.676, and hemoglobin was no longer an independent predictor, indicating that part of the observed model performance may have been influenced by the inclusion of transfusion in the composite endpoint. DeLong analysis demonstrated statistically significant differences compared with several established scores (*p* < 0.01). *Conclusions*: In this endoscopy-confirmed non-variceal UGIB cohort, the HOLD-B score demonstrated promising discriminative performance for predicting a clinically significant composite outcome using only emergency department variables. Because this composite outcome included management-related interventions in addition to clinical events, the model should not be interpreted as predicting individual clinical endpoints. Prospective external validation in broader emergency department populations is required before routine clinical implementation.

## 1. Introduction

Upper gastrointestinal bleeding (UGIB) remains a common emergency in emergency departments, and it continues to be linked to significant illness, death, and healthcare costs worldwide [1,2]. Quickly identifying patients at high risk is crucial for making triage decisions, determining the urgency of endoscopic procedures, and using hospital resources efficiently [3,4]. Several clinical scoring systems have been developed to help doctors quickly assess the risk of UGIB in patients. The Glasgow-Blatchford score (GBS) is widely used to predict the need for medical treatments like blood transfusions or endoscopic procedures [5]. Studies have shown that GBS is very good at identifying patients who need these treatments [6]. In addition, other scoring systems, such as the AIMS65 and Rockall scores, have been created to predict outcomes for patients with UGIB [7,8].

Although these scoring systems provide useful prognostic information, they also have limitations. Certain predictive models require the inclusion of numerous clinical variables, whereas others incorporate endoscopic observations that are typically absent during the initial emergency department assessment [6,8]. Pre-endoscopic scores, including the pre-endoscopic Rockall score, are widely used; however, their performance in predicting composite clinically significant outcomes in contemporary emergency department populations remains debated. Furthermore, more recent models, such as the ABC score and those employing machine-learning techniques, have sought to enhance prognostic accuracy; however, many still necessitate intricate computations or supplementary variables, potentially diminishing their utility in the time-constrained emergency department setting [9,10,11,12].

Because emergency physicians frequently must make rapid decisions with limited clinical information, there is a clear need for simpler and more practical risk-stratification tools. Accordingly, this study aimed to develop and internally validate a novel pre-endoscopic risk score in an endoscopy-confirmed non-variceal UGIB cohort using routinely available emergency department variables. This model, by facilitating earlier and more precise triage decisions, could aid prompt clinical intervention and more effective allocation of healthcare resources.

## 2. Materials and Methods

### 2.1. Study Design and Setting

This retrospective single-center prognostic model development study with internal validation was conducted in the Emergency Department of İzmir Atatürk Training and Research Hospital. The study included patients who visited the emergency department from January 2023 to January 2025. This tertiary care center sees about 360,000 to 400,000 patients annually. It provides care for a wide range of acute medical conditions. Ethical approval was obtained from the İzmir Katip Çelebi University Health Sciences Ethics Committee on 13 March 2025 (approval number: 0114). The study followed the Declaration of Helsinki. Given the retrospective design and the use of anonymized medical records, informed consent was not required. The study was retrospectively registered at ClinicalTrials.gov for transparency (Identifier: NCT07458178). Because this study involved a retrospective analysis of previously collected anonymized medical records, no prospective participant enrollment or intervention was performed.

### 2.2. Study Population

**Figure 1:**
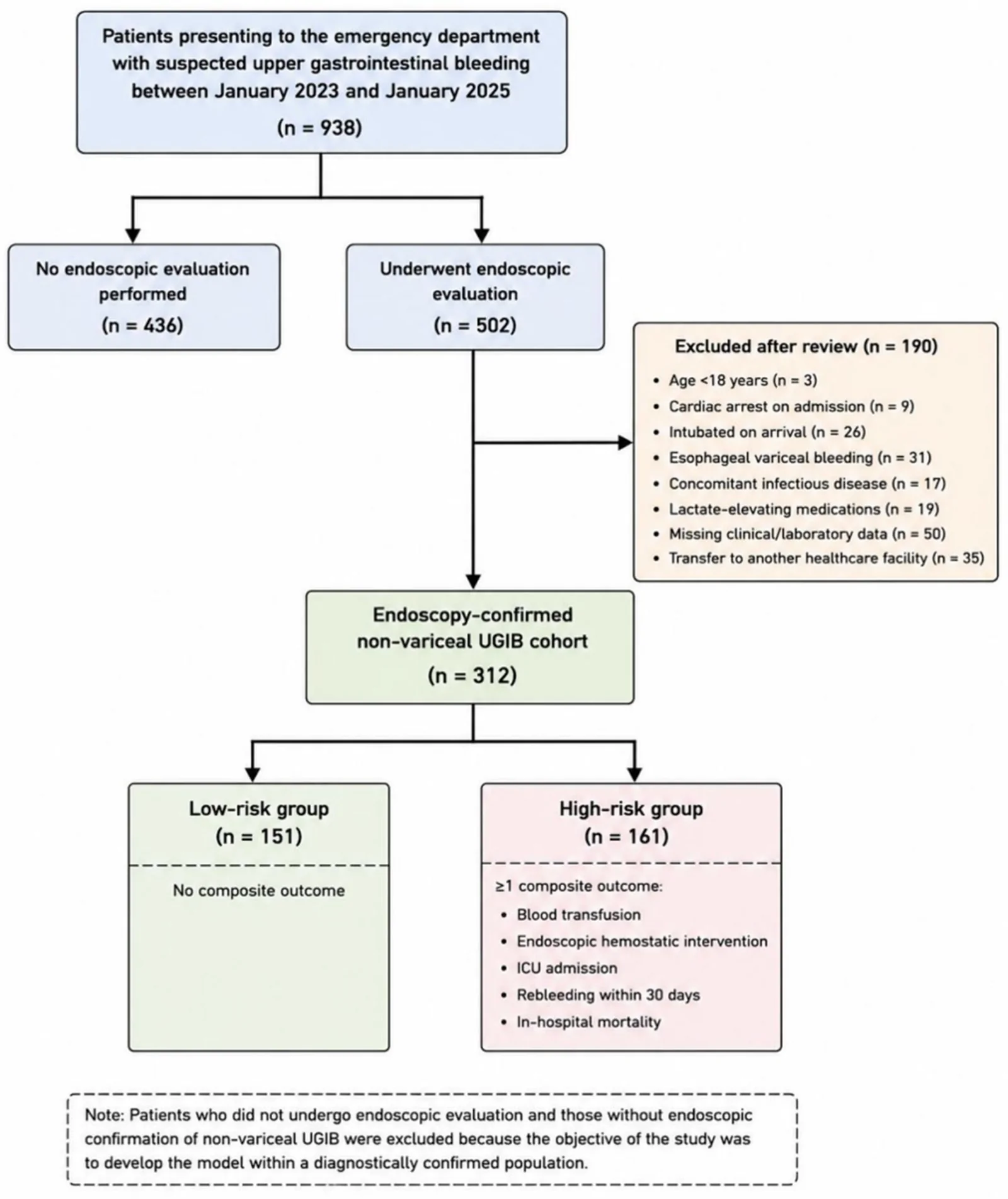
Flow diagram of patient selection and derivation of the endoscopy-confirmed non-variceal upper gastrointestinal bleeding cohort.

Patients aged 18 and older were included if they presented to the emergency department with hematemesis, melena, or hematochezia from a suspected upper GI origin with rapid transit, regardless of chief complaint. To ensure diagnostic accuracy, only patients who underwent endoscopic evaluation and had positive endoscopic findings compatible with non-variceal upper gastrointestinal bleeding were included in the analysis. Therefore, the derivation cohort represents a selected endoscopy-referred non-variceal UGIB population rather than an unselected emergency department UGIB population. Accordingly, the developed model should be interpreted within this derivation cohort. Ultimately, 312 patients were included in the study as described in the study flowchart (Figure 1).

Patients were excluded if they were under 18, presented with cardiac arrest, or arrived intubated. Patients with infectious diseases, esophageal variceal bleeding, or those using medications that increase lactate (such as metformin, beta-2 agonists, methotrexate, zidovudine, linezolid) were also excluded. Those without complete clinical or laboratory data, or whose outcomes were unclear due to transfer to another institution, were not included (Figure 1).

### 2.3. Data Collection and Variables

Study data were retrospectively obtained from the hospital information management system and electronic medical records. Demographic characteristics, presenting symptoms, vital signs, laboratory parameters, comorbid conditions, endoscopic findings, and clinical outcomes of the patients were recorded. Vital signs measured at admission to the emergency department and routine laboratory tests were documented. The candidate predictor variables evaluated for model development included presenting symptoms, admission vital signs (heart rate, systolic and diastolic blood pressure, respiratory rate, body temperature, oxygen saturation, and Glasgow Coma Scale), laboratory parameters (hemoglobin, white blood cell count, platelet count, blood urea nitrogen, creatinine, lactate, albumin, C-reactive protein, alanine aminotransferase, aspartate aminotransferase, international normalized ratio, and activated partial thromboplastin time), demographic characteristics, and relevant comorbid conditions available at emergency department presentation. Information regarding patients’ comorbid diseases, presenting clinical findings, and endoscopic evaluation results was also included in the dataset. Clinical outcomes included the need for blood transfusion, hospital admission, ICU admission, rebleeding, and length of hospital stay. Prognostic scores from the literature were calculated for each patient—these included the GBS, Rockall, AIMS65, and H3B2 scores. Derived biochemical ratios and the HOLD-B score were also analyzed.

### 2.4. Calculation of Risk Scores

Established upper gastrointestinal bleeding risk scores, including the Glasgow-Blatchford score (GBS), Rockall score, AIMS65, H3B2, and Pre-endoscopic Rockall score, were calculated according to their original published definitions. Only variables available at the time of emergency department presentation were used for pre-endoscopic scores. For scores requiring endoscopic findings, calculations were performed using documented endoscopic results. All scores were calculated retrospectively based on recorded clinical and laboratory data. In addition, the ABC score [9] and the ABL score [10], both recently proposed in the literature, were calculated according to their original definitions to evaluate their comparative performance within the present study population.

### 2.5. Definition of High-Risk and Low-Risk Groups

Patients were stratified into high- and low-risk groups based on a clinically relevant composite outcome, as described by Guner et al. [10]. A patient was classified as high risk if at least one of the following events occurred during hospitalization: requirement for blood transfusion, need for endoscopic hemostatic intervention (including clipping, injection therapy, band ligation, or thermal therapy), admission to the intensive care unit (ICU), rebleeding within 30 days, or in-hospital mortality. Patients who did not experience any of these events were classified as low risk. This composite outcome was selected to reflect clinically significant deterioration requiring escalation of care, encompassing both adverse clinical events and management-related interventions, consistent with previous studies.

### 2.6. Outcome Measures

#### 2.6.1. Primary Outcome

The primary outcome was the prediction of high-risk status in patients with upper gastrointestinal bleeding (UGIB). The study aimed to develop a novel prognostic scoring system to identify high-risk patients early.

#### 2.6.2. Secondary Outcomes

The secondary outcome was to compare the discriminative performance of the novel score with that of existing UGIB risk scores.

### 2.7. Statistical Analysis

All statistical analyses were performed using SPSS (IBM Corp., Armonk, NY, USA, version 26) and R-based Jamovi software (version 2.5.3). The distribution of continuous variables was assessed using the Kolmogorov–Smirnov test, along with histograms and Q–Q plots. Normally distributed variables were presented as mean ± standard deviation, whereas non-normally distributed variables were expressed as median and interquartile range (IQR). Comparisons between two independent groups were conducted using the independent-samples *t*-test when parametric assumptions were met and the Mann–Whitney U test when they were not. Categorical variables were expressed as counts and percentages (%), and group comparisons were performed using the Chi-square test or Fisher’s exact test when expected cell counts were less than five. Effect sizes were reported as odds ratios (OR) with 95% confidence intervals (CI). Odds ratios for continuous variables were calculated per one-unit increase in the respective variable. To identify independent predictors of high-risk status, univariate logistic regression analyses were initially performed, and variables with *p* < 0.10 were entered into the multivariable model. A binary logistic regression model was constructed using maximum likelihood estimation. Model assumptions were evaluated, and multicollinearity was assessed using variance inflation factors (VIFs); variables with VIF > 3 were excluded from the model. The overall model significance was evaluated using the Omnibus test, and calibration was assessed using the Hosmer–Lemeshow goodness-of-fit test.

Model explanatory power was reported using McFadden’s R^2^ coefficient. Continuous variables were categorized based on optimal cutoff values determined by receiver operating characteristic (ROC) curve analysis to enhance clinical interpretability. Discriminative performance was evaluated using ROC curve analysis, and the area under the curve (AUC) with 95% confidence intervals was calculated. Optimal cutoff values were determined using the Youden index (J = sensitivity + specificity − 1). Diagnostic performance metrics, including sensitivity, specificity, positive predictive value (PPV), negative predictive value (NPV), positive likelihood ratio (+LR), and negative likelihood ratio (−LR), were calculated. The discriminative performance of the newly developed HOLD-B score was compared with Rockall, Glasgow-Blatchford (GBS), AIMS65, H3B2, ABC, ABL, and pre-endoscopic Rockall (Pre-RS) scores. Differences between AUC values were analyzed using the DeLong method. Internal model validation was performed using bootstrap resampling with 1000 iterations, followed by the computation of optimism-corrected AUC values. Calibration plots were then utilized to evaluate model calibration, focusing on the alignment between observed and predicted probabilities. Furthermore, Decision Curve Analysis (DCA) was performed to assess clinical utility, with net clinical benefit evaluated across a range of threshold probabilities. To determine the clinical applicability of the scoring system, classification accuracy and misclassification rates across distinct risk categories were analyzed. Missing data were handled using a complete-case approach. Only patients with complete data for all candidate predictor variables were included in the final analysis, and no imputation methods were applied. The adequacy of the sample size for binary logistic regression was evaluated using the Peduzzi approach (N ≥ _10 k/p), thereby confirming the sufficiency of the available sample size relative to the number of variables incorporated within the model. All analyses were conducted using a two-tailed approach, and a *p*-value of less than 0.05 was deemed statistically significant.

## 3. Results

Among the 312 patients included in the study, 51.6% (n = 161) were classified as high risk. Compared with the low-risk group, patients in the high-risk group had significantly higher heart rate and respiratory rate, and significantly lower systolic and diastolic blood pressures at admission (all *p* < 0.05). Among the clinical symptoms, melena (*p* = 0.035) and evidence of active bleeding (*p* < 0.001) were significantly associated with high-risk classification. Hemoglobin and albumin levels were significantly lower in the high-risk group, whereas lactate, BUN, lactate/hemoglobin ratio, and BUN/Creatinine ratio were significantly higher. Length of hospital stay was also significantly longer in the high-risk group (Table 1). The high odds ratio observed for the lactate/hemoglobin ratio reflects the effect estimate per one-unit increase in the ratio, which has a limited numerical range.

Clinical outcomes are summarized in Table 2. As expected, these outcome variables were more frequent in the high-risk group because they constituted the predefined composite endpoint and were therefore not included among baseline characteristics.

In the ROC analysis of the independent predictors, hemoglobin ≤8.05 g/dL and BUN >44.5 mg/dL yielded the highest AUCs (both AUCs = 0.711). Hemoglobin ≤8.05 g/dL showed 57.8% sensitivity and 79.5% specificity, whereas BUN > 44.5 mg/dL showed 47.2% sensitivity and 85.4% specificity. A diastolic blood pressure threshold of ≤67.5 mmHg predicted high-risk status with 62.1% sensitivity (AUC = 0.674), while lactate >2.15 mmol/L showed 81.5% specificity (AUC = 0.626). Among the evaluated predictors, BUN had the highest positive likelihood ratio (+LR = 3.24) (Table 3). The identical AUC value of 0.711 for hemoglobin and BUN was re-checked against the source dataset and confirmed to be a genuine coincidental result rather than a transcription or formatting error.

The binary logistic regression model constructed to identify independent predictors of high-risk status was statistically significant (McFadden’s R^2^ = 0.219; N = 312). Hemoglobin ≤8.05 g/dL, BUN > 44.5 mg/dL, lactate >2.15 mmol/L, and diastolic blood pressure ≤67.5 mmHg were identified as independent predictors of high-risk classification. Hemoglobin ≤8.05 g/dL was associated with a 4.19-fold increase in the odds of high-risk status (OR = 4.191, *p* < 0.001), while BUN > 44.5 mg/dL was associated with a 3.39-fold increase (OR = 3.390, *p* < 0.001). Lactate >2.15 mmol/L and diastolic blood pressure ≤67.5 mmHg were associated with 2.75-fold and 1.99-fold increases in the odds of high-risk status, respectively. Based on the regression coefficients, the HOLD-B scoring system was developed, assigning 3 points each to hemoglobin and lactate, and 2 points each to BUN and diastolic blood pressure, yielding a total score ranging from 0 to 10 (Table 4).

**Figure 2:**
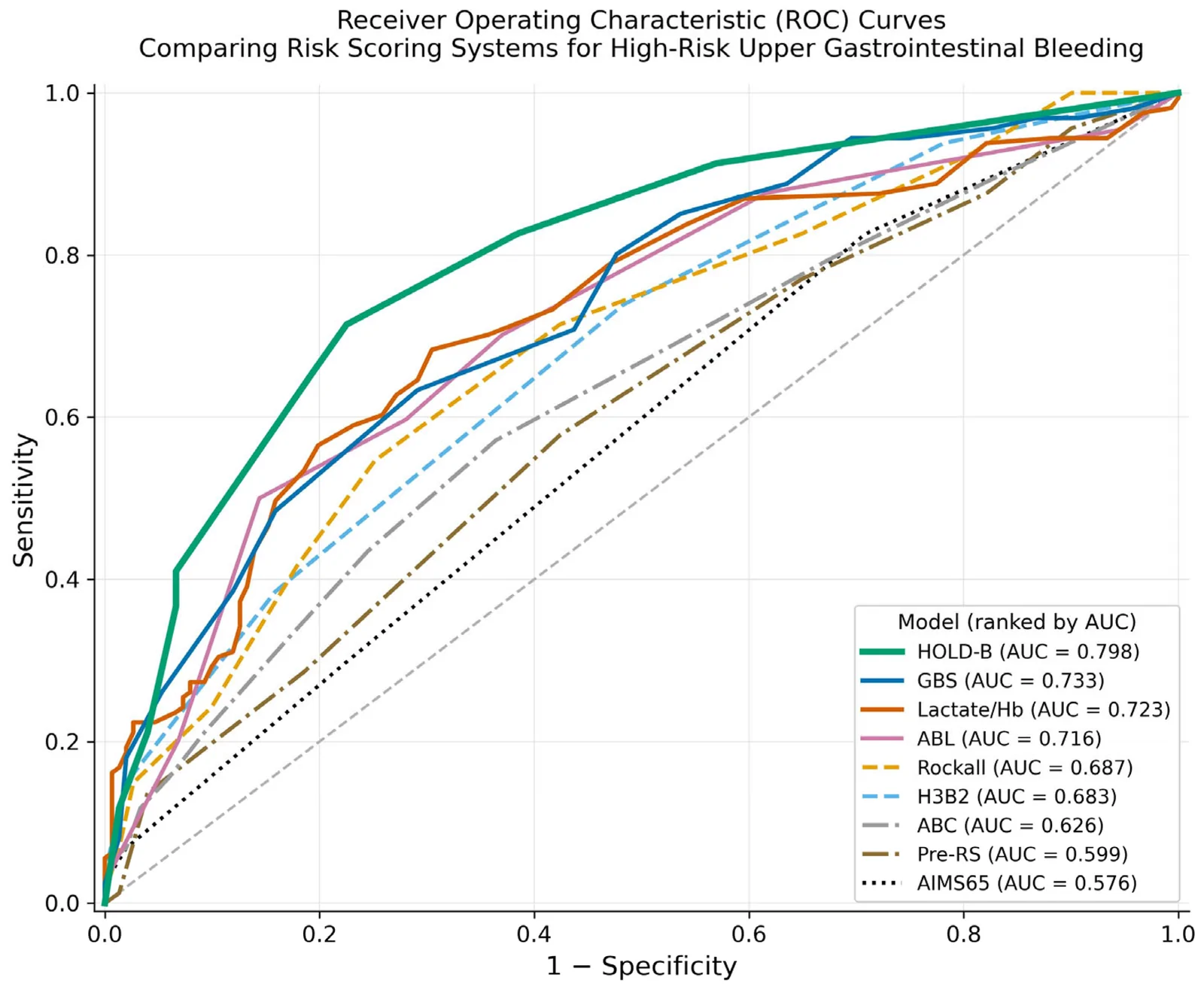
Receiver Operating Characteristic (ROC) Curves Comparing the Discriminative Performance of Risk Scoring Systems for Predicting High-Risk Upper Gastrointestinal Bleeding.

Figure 2 shows the ROC curves of the evaluated risk scoring systems for predicting high-risk upper gastrointestinal bleeding. Visually, the HOLD-B score showed the greatest separation from the reference line, consistent with its highest AUC value among the evaluated scores.

Among the evaluated risk-scoring systems, the HOLD-B score achieved the highest AUC (0.798; 95% CI: 0.749–0.846), indicating good discriminative performance. The HOLD-B score showed higher AUC values than several commonly used scoring systems, including GBS, ABL, and Rockall. In contrast, AIMS65 (AUC = 0.576) and the Pre-endoscopic Rockall Score (AUC = 0.599) demonstrated the lowest performance in risk prediction. The optimal cutoff value of 3.5 (≥4) for the HOLD-B score yielded balanced and effective clinical performance in identifying high-risk patients, with 71.4% sensitivity and 77.4% specificity. The HOLD-B score also yielded the lowest negative likelihood ratio (−LR = 0.369) among the evaluated scoring systems. As a single parameter, the Lactate/Hb ratio (AUC = 0.723) also demonstrated a higher diagnostic performance than several complex scoring systems, including ABC, Pre-RS, and AIMS65 (Table 5).

Pairwise AUC comparisons performed using the DeLong test demonstrated significant differences in discriminative performance among the scoring systems. The HOLD-B score showed significantly higher discriminative power compared with the ROCKALL (ΔAUC = −0.110, *p* < 0.001), GBS (ΔAUC = −0.065, *p* = 0.008), AIMS65 (ΔAUC = −0.221, *p* < 0.001), H3B2 (ΔAUC = −0.115, *p* < 0.001), ABC (ΔAUC = −0.172, *p* < 0.001), and PRE-RS (ΔAUC = −0.198, *p* < 0.001) scores. These findings indicate that the HOLD-B score demonstrated significantly higher discriminative performance than several established scoring systems for identifying high-risk patients. The GBS demonstrated significantly higher AUC values than the AIMS65 (*p* < 0.001), ABC (*p* < 0.001), PRE-RS (*p* < 0.001), and H3B2 (*p* = 0.011) scores, while no significant difference was found between the GBS and ABL scores (*p* = 0.628). Although the AIMS65 score is primarily designed as a mortality-focused scoring system, it showed lower discriminative performance compared with several other scoring systems. It was significantly weaker than the HOLD-B score (ΔAUC = −0.221, *p* < 0.001). Similarly, the PRE-RS score demonstrated significantly lower performance compared with the HOLD-B score (*p* < 0.001).

The Lactate/Hb ratio, a single biochemical parameter, performed comparably to some classical scores; however, its discriminatory power was significantly lower than that of the HOLD-B score (ΔAUC = 0.074, *p* < 0.001). Overall, the HOLD-B score demonstrated greater discriminative capacity than both complex clinical scoring systems and single-biomarker-based approaches in this study population. These findings suggest that the developed score may represent a promising tool for the early and accurate classification of high-risk patients with upper gastrointestinal bleeding in clinical practice (Table 6).

### Additional Validation and Sensitivity Analyses

Sensitivity analysis excluding transfusion from the composite endpoint reduced HOLD-B’s AUC from 0.798 to 0.676. While hemoglobin lost independent predictive value, lactate and BUN remained significant predictors (Appendix A Table A3, Table A5, Table A7 and Table A8; Appendix B Figure A4 and Figure A6).

**Figure A3:**
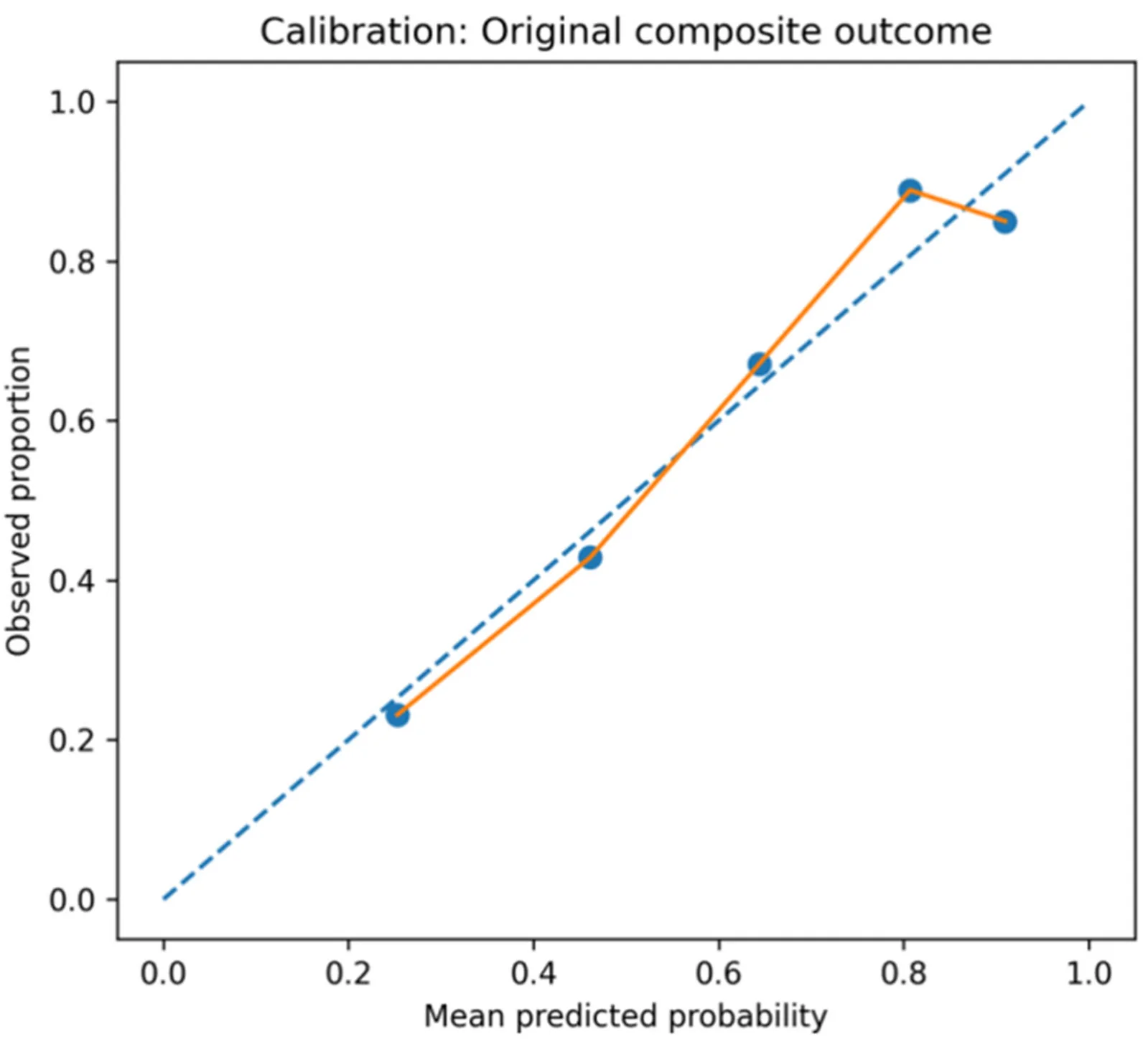
Calibration Chart for Original Composite endpoint.

**Figure A4:**
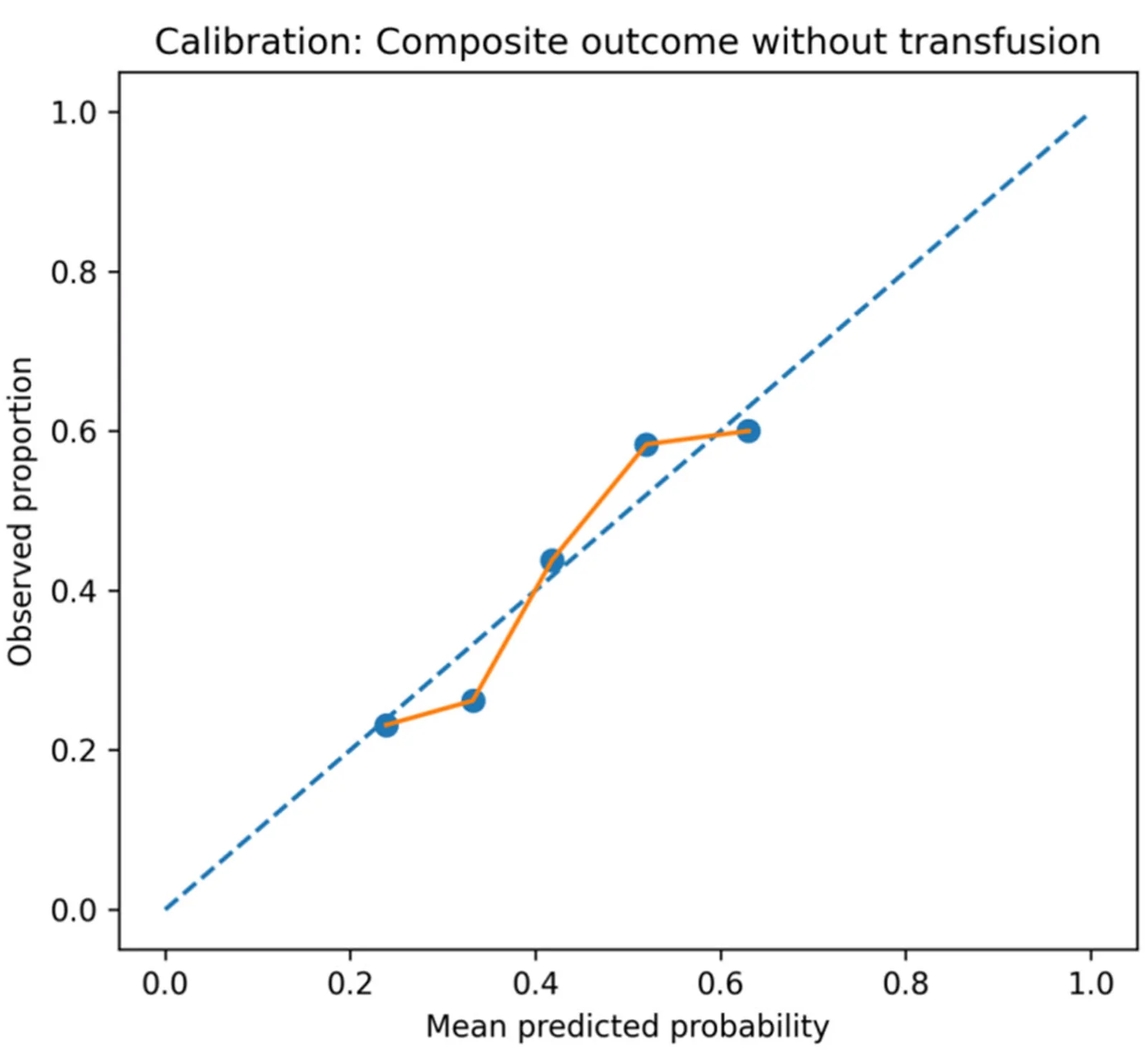
Calibration Chart for Transfusion-excluded composite endpoint.

Internal validation using 1000 bootstrap resamples demonstrated minimal optimism. The apparent AUC of the HOLD-B model was 0.802, and the optimism-corrected AUC was 0.796, corresponding to an average optimism of 0.006 (Appendix A Table A8). Calibration was acceptable (Hosmer–Lemeshow χ^2^ = 5.108, *p* = 0.530) (Appendix A Table A8; Appendix B Figure A3).

**Figure A5:**
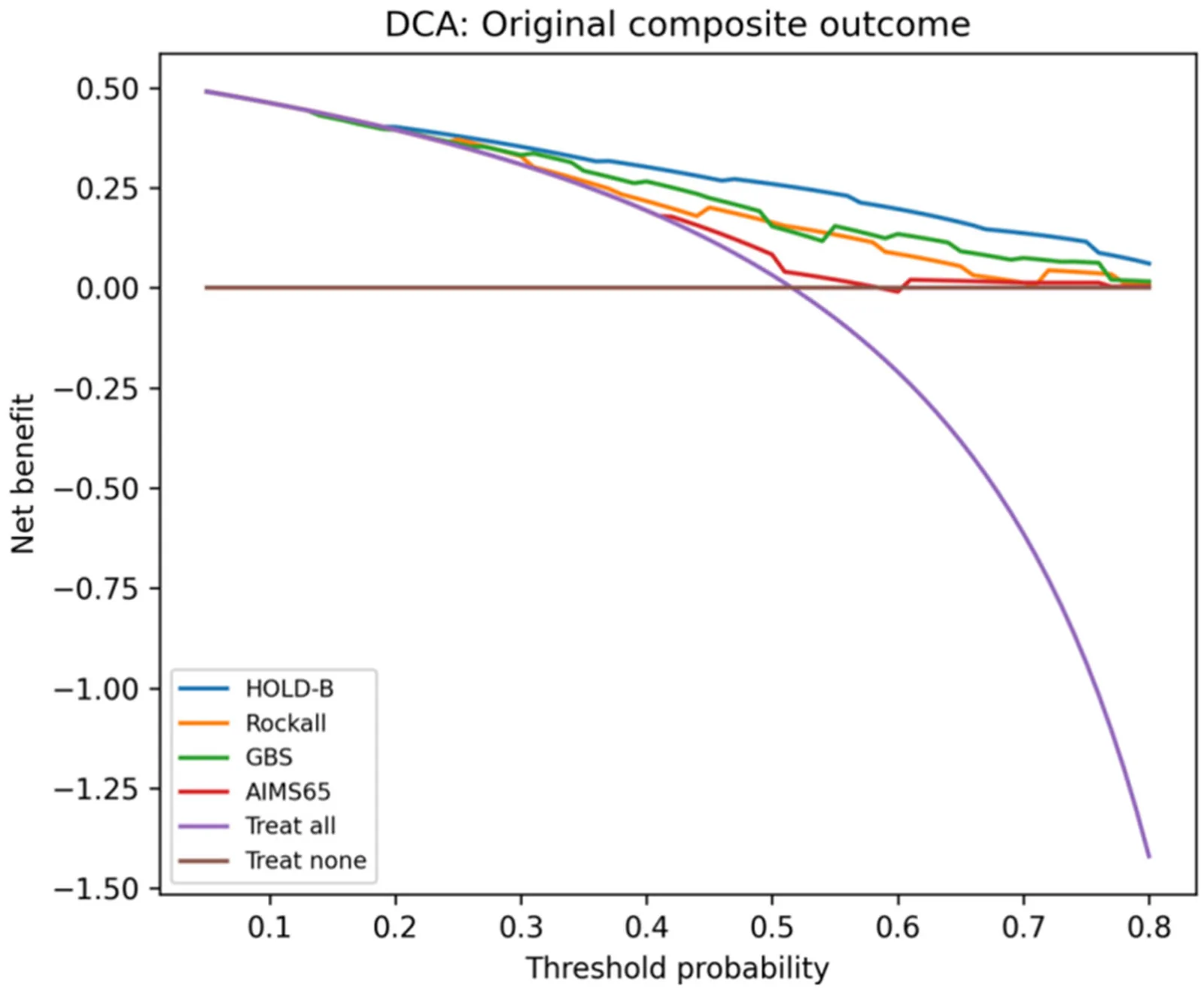
Decision Curve Analysis for the Original Composite endpoint.

**Figure A6:**
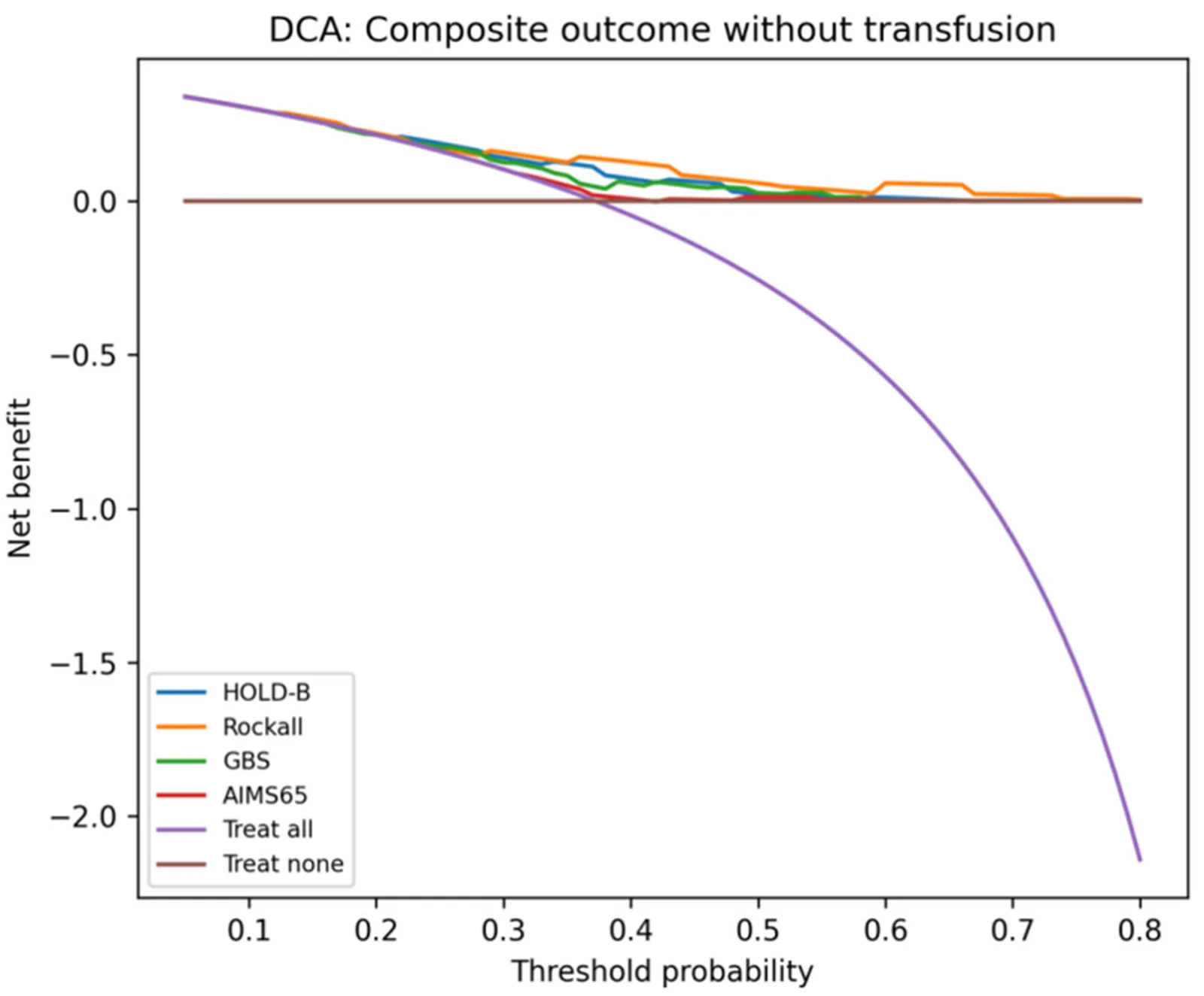
Decision Curve Analysis for Transfusion-excluded composite endpoint.

Calibration plots demonstrated good agreement between predicted and observed probabilities. Decision curve analysis showed a favorable net clinical benefit across clinically relevant threshold probabilities (Appendix B Figure A5).

## 4. Discussion

Acute UGIB is common in emergency departments and causes significant morbidity, mortality, and healthcare resource use [1,2,3,4]. Identifying high-risk patients quickly informs triage protocols, determines the need for urgent endoscopic procedures, and optimizes hospital resources. To stratify clinical risk, the GBS, AIMS65, and Rockall scores were developed [5,6,7,8]. Despite their widespread use, predictive accuracy and clinical utility have varied across patient cohorts and clinical endpoints [11,13,14]. These issues highlight the need for simple, physiologically relevant risk-stratification tools for initial assessment in the emergency department.

The present study should be interpreted within the context of its derivation cohort. Unlike studies including all patients presenting to the emergency department with suspected UGIB, our model was derived from patients with endoscopy-confirmed non-variceal UGIB. Therefore, the observed performance reflects this selected population and should not be extrapolated to all emergency department presentations without external validation.

We created the HOLD-B score using hemoglobin, lactate, BUN, and diastolic blood pressure from emergency department presentations. The HOLD-B score had an AUC of 0.798 and could identify high-risk patients with UGIB. This performance outperformed the GBS (AUC = 0.733), ABL (0.716), and Rockall (0.687) scores. These findings suggest that combining physiologically relevant variables may improve the assessment of acute UGIB risk.

Recent studies have proposed the ABL score as a simplified tool for risk stratification in acute upper gastrointestinal bleeding. Incorporating albumin, BUN, and lactate, this score has been reported to demonstrate moderate discriminative performance in predicting clinically significant high-risk outcomes among patients with upper gastrointestinal bleeding presenting to the emergency department [10]. ABL scores measure metabolic markers related to bleeding severity, not blood pressure. Metabolic markers and diastolic blood pressure help the HOLD-B score detect circulatory issues earlier. This may explain why our study group distinguishes outcomes better. Together, the physiological perspectives on the HOLD-B score may explain its improved effectiveness.

Hemoglobin concentration, an indicator of blood loss severity, has been linked to increased transfusion needs and poor prognoses in gastrointestinal bleeding [15]. In the early stages, the body can temporarily maintain hemoglobin levels through compensatory processes. In UGIB, hemoglobin serves as both a prognostic indicator and a criterion for transfusion [16]. Evidence and guidelines support restrictive transfusion practices (hemoglobin ~7–8 g/dL, marginally higher in cases of cardiac disease), emphasizing hemodynamic stability and active hemorrhage over achieving a normal hemoglobin level, as more liberal transfusions exacerbate rebleeding and may elevate mortality rates [17]. In HOLD-B, hemoglobin also has a strong effect on the scoring system, with a cutoff value of 8.05. Given that a decrease in hemoglobin can be compensated for by sympathetic activation and thus mask early findings, changes in diastolic blood pressure may provide more reliable prognostic information than changes in systolic blood pressure. Because baseline hemoglobin concentration is an important determinant of transfusion decisions, incorporation bias cannot be excluded. In our sensitivity analysis, exclusion of blood transfusion from the composite endpoint reduced the AUC from 0.798 to 0.676, and hemoglobin was no longer an independent predictor. These findings suggest that part of the observed predictive performance may have been influenced by the inclusion of transfusion in the composite endpoint, in addition to reflecting bleeding severity. Nevertheless, lactate, BUN, and diastolic blood pressure remained significant contributors to the overall model performance, supporting the multifactorial nature of the HOLD-B score.

Serum lactate levels indicate metabolic stress and tissue blood flow. When blood volume suddenly decreases, the body receives less oxygen. Consequently, lactate levels climb, a direct result of heightened anaerobic metabolism. Elevated lactate concentrations have been associated with poorer prognoses in cases of gastrointestinal hemorrhage and other severe medical conditions [18]. In our study group, the lactate-to-hemoglobin ratio was a predictor of both bleeding intensity and systemic hypoperfusion. Nevertheless, the demonstrated higher discrimination in our cohort compared to this single parameter implies that incorporating physiological markers could provide a more comprehensive assessment of bleeding severity.

In the HOLD-B model, diastolic blood pressure is a key factor. A 2024 ED model for forecasting ICU admission in UGIB identified SBP and DBP as significant independent predictors, along with hemoglobin, platelets, ALT, and PT. Yang et al. [19] indicate that elevated diastolic blood pressure (DBP) correlates with increased odds of ICU admission, which they interpret as an indicator of compensatory vasoconstriction during early shock. In a study by Deng et al. [20], elderly patients showed an increase in DBP during early endoscopy, followed by a decline to pre-procedure levels. Moreover, they describe that after the procedure, the elderly exhibit a more pronounced decrease in diastolic blood pressure, indicating reduced reserve and volume sensitivity. In non-elderly UGIB patients, they found that DBP is more stable before and after intervention. To our knowledge, diastolic blood pressure alone has not been incorporated as a primary variable in established UGIB risk scores. In this respect, HOLD-B differs from existing UGIB risk scores. The body’s sympathetic nervous system might temporarily maintain systolic pressure stability, even as diastolic pressure drops [21]. Diastolic blood pressure may indicate circulatory compromise before current risk-assessment tools, which may contribute to the clinical relevance of HOLD-B.

Numerous studies indicate that elevated BUN at presentation, specifically BUN levels exceeding 20 mg/dL [22], significantly high BUN (≥130 mg/dL) [23], or an increase in BUN over 24 h in cases of UGIB, are consistently correlated with more severe hemorrhage, an increased likelihood of rebleeding, the necessity for intervention, and higher mortality rates. Several UGIB risk assessment tools (e.g., GBS, AIMS65) include BUN (urea) as a fundamental variable [24,25]. Similarly, in HOLD-B, BUN is an important component, with a cutoff of 44.5, and may reflect the systemic response to bleeding-related hypovolemia and protein breakdown.

The favorable performance of HOLD-B likely reflects its ability to capture key physiological manifestations of acute bleeding within this endoscopy-referred non-variceal UGIB cohort. Several clinical and laboratory variables are needed to predict clinical interventions using the GBS [5]. Albumin levels and altered mental status may indicate long-term health issues rather than bleeding severity, even though the AIMS65 score predicts mortality [7]. The Rockall score includes endoscopic findings, making it less useful in the ED before endoscopy [8,26]. The HOLD-B score works to overcome these practical limitations and provide a model that uses only physiological parameters at emergency department presentation. Since existing scores predict different clinical outcomes and were derived in different patient populations, the observed higher discriminatory performance of HOLD-B should be interpreted within the context of the selected composite endpoint and the present derivation cohort rather than as evidence of universal superiority across all UGIB-related clinical settings. The composite endpoint comprised both clinical outcomes (mortality and rebleeding) and management-related interventions (blood transfusion, endoscopic hemostasis, and ICU admission), which are not clinically equivalent but were selected to represent overall clinically significant deterioration requiring escalation of care, consistent with previous studies. Furthermore, because blood transfusion was included in the composite endpoint and hemoglobin is one of its components, part of the observed performance may have been influenced by this outcome definition.

Machine learning models and artificial neural networks have outperformed traditional scoring systems, such as the Glasgow-Blatchford, Rockall, and AIMS65 scores, in multiple studies, with reported AUC values ranging from 0.84 to 0.93 across various cohorts [12,27]. These values are higher than the discriminatory performance observed for the HOLD-B score in our study. Age, comorbidities, hemodynamic parameters, and routine laboratory markers such as hemoglobin, BUN, albumin, platelets, and coagulation indices have been identified as strong predictors in these models [28]. However, such approaches require complex computational infrastructure, which may limit their practical applicability in the fast-paced emergency medicine setting. Although machine learning models are more recent than conventional techniques, they rely on algorithms capable of simultaneously evaluating numerous variables. Within the evolving landscape of machine learning, HOLD-B may be considered a robust and practical risk-stratification tool in settings where advanced computational systems are unavailable or rapid clinical decision-making is required.

Precise risk stratification facilitates expedited endoscopic intervention, enhances patient prioritization, and optimizes resource allocation. The HOLD-B score, which can be easily calculated at the bedside using data from the initial emergency department assessment, avoids the need for complex calculations or endoscopic procedures. Our study’s results showed that a cutoff value of 3.5 (≥4) provided the best balance between sensitivity and specificity. Patients with a HOLD-B score ≤3 can be considered low-risk. However, patients with a score ≥4 can be considered high risk and require vigilance, close monitoring, and early follow-up or intervention. Although it is premature to make strong recommendations regarding the widespread adoption of the HOLD-B scoring system, its ease of application and the incorporation of diastolic blood pressure—without a systolic component—for the first time in a UGIB risk score suggest that it may serve as a valuable tool in clinical practice.

### Limitations

Several limitations should be acknowledged. First, the single-center and retrospective design may limit generalizability and introduce selection bias. The study population was restricted to patients who underwent endoscopy and had positive endoscopic findings; therefore, patients discharged directly from the emergency department, patients not referred for endoscopy, and patients with negative endoscopic findings were not represented. This selection strategy may have increased the prevalence of adverse outcomes and may limit extrapolation to broader emergency department UGIB populations. In addition, missing data were handled using a complete-case approach; therefore, selection bias cannot be ruled out if the missingness was not completely random.

Second, retrospective data acquisition may have introduced measurement variability, particularly for vital signs and laboratory parameters such as lactate. In addition, the composite endpoint comprised clinically heterogeneous components: transfusion, endoscopic intervention, ICU admission, rebleeding, and mortality. Sensitivity analysis excluding transfusion demonstrated reduced discriminatory performance, suggesting that incorporation bias may have contributed to the primary model performance (Appendix A Table A3, Table A5 and Table A7).

Third, although bootstrap resampling was used for internal validation, this approach does not replace external validation in independent cohorts. In addition, model calibration was evaluated using the Hosmer–Lemeshow goodness-of-fit test and calibration plots; however, the calibration slope and calibration intercept were not separately estimated, which may have provided a more comprehensive assessment of calibration performance. The exclusion of variceal bleeding and patients using lactate-altering medications may also limit generalizability, particularly in populations with a high prevalence of liver disease. Therefore, the HOLD-B score should be considered a supplementary risk stratification tool, and prospective multicenter external validation studies are required before routine clinical use.

## 5. Conclusions

In this endoscopy-referred non-variceal UGIB cohort, the HOLD-B score provided a simple, physiologically grounded approach to early risk stratification. By integrating markers of hemorrhage severity, metabolic response, and hemodynamic instability, the model demonstrated promising discriminatory performance within the selected composite outcome and compared favorably with several commonly used pre-endoscopic risk scores. Because blood transfusion was included as a component of the composite endpoint, part of the observed model performance may have been influenced by the outcome definition. Therefore, the HOLD-B score should be interpreted as predicting a clinically significant composite outcome rather than any individual clinical endpoint. Prospective multicenter external validation in broader emergency department populations, including patients without endoscopic confirmation, is required before routine clinical implementation.

## Figures and Tables

**Table 1:** Baseline characteristics of the study population according to risk group.

Variables	Low Risk (n = 151)	High Risk (n = 161)	*p*	OR
Age	69.66 ± 14.82	70.52 ± 15.78	0.621	1.004
Gender			0.083	1.495
Female	68 (45%)	57 (35.4%)		
Male	83 (55%)	104 (64.6%)		
Presenting Complaint			0.159	
Melena	57 (37.7%)	68 (42.2%)		
Hematemesis	46 (30.5%)	49 (30.4%)		
Hematochezia	45 (29.8%)	36 (22.4%)		0.893
Abdominal Pain	1 (0.7%)	4 (2.5%)		0.671
Dizziness	1 (0.7%)	0 (0.0%)		3.353
Fatigue	0 (0.0%)	2 (1.2%)		
Syncope	0 (0.0%)	2 (1.2%)		
Other	1 (0.7%)	0 (0.0%)		
Heart Rate	86.11 ± 14.73	90.21 ± 20.14	0.040	1.013
Systolic Blood Pressure	123.91 ± 26.19	114.35 ± 26.27	<0.001	0.986
Diastolic Blood Pressure	72.09 ± 11.87	64.39 ± 14.68	<0.001	0.958
Respiratory Rate	16.14 ± 2.20	18.25 ± 5.43	<0.001	1.258
Body Temperature	36.43 ± 0.35	36.45 ± 0.34	0.477	1.270
Oxygen Saturation	96.90 ± 1.40	95.99 ± 2.58	<0.001	0.775
Glasgow Coma Scale	14.91 ± 0.44	14.80 ± 0.61	0.077	0.667
Lactate (mmol/L)	1.66 ± 0.88	2.38 ± 1.86	<0.001	1.582
Hemoglobin	10.00 ± 2.18	8.14 ± 2.80	<0.001	0.749
WBC	10,169.54 ± 14,124.63	12,807.85 ± 13,616.96	0.094	1.000
PLT	249,543.05 ± 116,765.85	239,006.21 ± 118,492.06	0.430	1.000
BUN	29.60 ± 15.44	45.37 ± 23.80	<0.001	1.044
Creatinine	1.20 ± 1.04	1.26 ± 0.72	0.557	1.081
INR	1.76 ± 2.29	1.98 ± 3.18	0.480	1.030
aPTT	30.91 ± 8.58	29.93 ± 10.08	0.444	0.989
ALT	15.28 ± 8.89	19.48 ± 23.14	0.034	1.017
AST	24.24 ± 19.87	33.65 ± 44.92	0.016	1.009
Albumin	35.28 ± 5.29	31.57 ± 5.29	<0.001	0.896
CRP	19.10 ± 31.39	29.29 ± 46.83	0.024	1.007
Base Deficit	0.85 ± 4.21	1.88 ± 4.43	<0.001	1.058
Length of Hospital Stay (Days)	3.97 ± 2.71	6.38 ± 5.84	<0.001	1.194
Lactate/Hemoglobin Ratio	0.17 ± 0.11	0.33 ± 0.32	<0.001	282.388
INR/Albumin Ratio	0.05 ± 0.06	0.07 ± 0.14	0.097	10.020
BUN/Creatinine Ratio	27.90 ± 12.87	37.86 ± 16.56	<0.001	1.049
Presence of Hematemesis			0.366	1.241
Yes	48 (31.8%)	59 (36.6%)		
No	103 (68.2%)	102 (63.4%)		
Presence of Melena			0.035	1.700
Yes	99 (65.6%)	123 (76.4%)		
No	52 (34.4%)	38 (23.6%)		
Presence of Syncope			0.067	5.840
Yes	1 (0.7%)	6 (3.7%)		
No	150 (99.3%)	154 (95.7%)		
Hypertension			0.824	1.050
Yes	75 (49.7%)	82 (50.9%)		
No	76 (50.3%)	79 (49.1%)		
Diabetes Mellitus			0.944	1.020
Yes	37 (24.5%)	40 (24.8%)		
No	114 (75.5%)	121 (75.2%)		
COPD			0.073	2.270
Yes	7 (4.6%)	16 (9.9%)		
No	144 (95.4%)	145 (90.1%)		
Liver Disease			0.082	2.000
Yes	10 (6.6%)	20 (12.4%)		
No	141 (93.4%)	141 (87.6%)		
Heart Failure			0.144	1.740
Yes	12 (7.9%)	21 (13%)		
No	139 (92.1%)	140 (87%)		
Renal Failure			0.753	1.160
Yes	9 (6%)	11 (6.8%)		
No	142 (94%)	150 (93.2%)		
Coronary Artery Disease			0.795	1.070
Yes	43 (28.5%)	48 (29.8%)		
No	108 (71.5%)	113 (70.2%)		
Malignancy			0.297	1.400
Yes	19 (12.6%)	27 (16.8%)		
No	132 (87.4%)	134 (83.2%)		
Upper Gastrointestinal Malignancy			0.937	0.937
Yes	3 (2%)	3 (1.9%)		
No	148 (98%)	158 (98.1%)		
Endoscopy Findings			0.131	
Mallory-Weiss/Esophagitis	6 (4%)	14 (8.7%)		
Ulcer/Tumor/Erosion	143 (94.7%)	142 (88.2%)		0.426
Upper Gastrointestinal Malignancy	2 (1.3%)	5 (3.1%)		1.071
Rockall Score	4.35 ± 2.12	5.91 ± 2.30	<0.001	1.370
GBS	7.75 ± 4.02	11.06 ± 3.62	<0.001	1.250
AIMS65 Score	0.95 ± 0.76	1.22 ± 0.89	0.004	1.491
H3B2 Score	1.44 ± 1.04	2.25 ± 1.19	<0.001	1.898
ABC Score	3.03 ± 2.49	4.38 ± 3.17	<0.001	1.184
ABL Score	2.42 ± 1.67	3.86 ± 1.86	<0.001	1.540
Pre-RS	3.03 ± 1.63	3.61 ± 1.63	0.002	1.244

OR: Odds Ratio, WBC: White blood cell, PLT: Platelet, BUN: Blood urea nitrogen, INR: International normalized ratio, aPTT: Activated Partial Thromboplastin Time, ALT: Alanine Aminotransferase, AST: Aspartate Aminotransferase, CRP: C-reactive Protein, COPD: Chronic Obstructive Pulmonary Disease.

**Table 2:** Clinical outcomes of the study population.

Variables	n (%)
Evidence of Active Bleeding	79 (25.3)
Endoscopic Intervention	79 (25.3)
Blood Transfusion	88 (28.2)
Transfusion Unit	
0	224 (71.8)
1	13 (4.2)
2	45 (14.4)
3	26 (8.3)
4	4 (1.3)
Hospitalization admission	305 (97.8)
Intensive care unit admission	75 (24)
Rebleeding within 30 days	9 (2.9)
Discharged alive	294 (94.2)
In-hospital mortality	18 (5.8)

**Table 3:** Optimum cutpoint values obtained by ROC analysis of independent risk variables.

Variables	Optimum Cutpoint	Sensitivity (%)	Specificity (%)	PPV (%)	NPV (%)	AUC (95% CI)	+LR	−LR
DBP	67.5	62.1	66.2	66.2	62.1	0.674 (0.614–0.733)	1.839	0.572
Lactate	2.15	44.1	81.5	71.7	57.7	0.626 (0.564–0.689)	2.378	0.686
Hemoglobin	8.05	57.8	79.5	75	63.8	0.711 (0.653–0.768)	2.814	0.531
BUN	44.5	47.2	85.4	77.6	60.3	0.711 (0.654–0.765)	3.24	0.618

PPV: Positive predictive value, NPV: Negative predictive value, LR: Likelihood ratio, DBP: Diastolic blood pressure, BUN: Blood urea nitrogen.

**Table 4:** Multivariable logistic regression analysis for prediction of high-risk status and derivation of the HOLD-B score.

						CI 95%		
Variables	B	SE	Wald	*p*	OR	Lower	Upper	Score
Constant	−1.465	0.228	41.08	<0.001	0.231	0.148	0.362	-
DBP	0.689	0.270	6.50	0.011	1.992	1.173	3.384	
≤67.5								2
>67.5								0
Lactate	1.012	0.298	11.56	<0.001	2.751	1.535	4.930	
>2.15								3
≤2.15								0
Hemoglobin	1.433	0.280	26.11	<0.001	4.191	2.420	7.259	
≤8.05								3
>8.05								0
BUN	1.221	0.307	15.84	<0.001	3.390	1.858	6.187	
>44.5								2
≤44.5								0

CI: Confidence Interval, SE: Standard Error, OR: Odds Ratio, DBP: Diastolic blood pressure, BUN: Blood urea nitrogen.

**Table 5:** ROC Curve Comparison of Scores.

Score	Optimum Cutpoint	Sensitivity (%)	Specificity (%)	PPV (%)	NPV (%)	AUC (95% CI)	+LR	−LR
Rockall	5.5	54.7	74.8	69.84	60.75	0.687 (0.629–0.745)	2.172	0.606
GBS	10.5	82.6	29.1	69.86	64.46	0.733 (0.678–0.788)	2.174	0.517
AIMS65	0.5	73.9	51.7	55.42	61.11	0.576 (0.518–0.635)	1.166	0.597
H3B2	1.5	73.91	51.66	61.98	65.00	0.683 (0.627–0.739)	1.529	0.505
ABC	3.5	57.14	63.58	62.59	58.18	0.626 (0.564–0.687)	1.569	0.674
ABL	4.5	50.00	85.62	78.57	61.88	0.716 (0.659–0.774)	3.476	0.584
PRE-RS	3.5	57.76	57.62	59.24	56.13	0.599 (0.538–0.661)	1.363	0.733
HOLD-B	3.5	71.43	77.48	77.18	71.78	0.798 (0.749–0.846)	3.172	0.369
Lactate/Hb	0.175	68.32	69.54	70.51	67.31	0.723 (0.667–0.780)	2.243	0.456

PPV: Positive predictive value, NPV: Negative predictive value, LR: Likelihood ratio.

**Table 6:** Pairwise Comparisons of AUC Values Using DeLong’s Test.

Comparison	ΔAUC	95% CI	z	*p*
ROCKALL vs. GBS	−0.046	[−0.106, 0.015]	−1.47	0.142
ROCKALL vs. AIMS65	0.111	[0.048, 0.174]	3.47	<0.001
ROCKALL vs. H3B2	0.004	[−0.057, 0.065]	0.13	0.894
ROCKALL vs. ABC	0.062	[0.014, 0.109]	2.53	0.011
ROCKALL vs. ABL	−0.029	[−0.101, 0.043]	−0.80	0.426
ROCKALL vs. PRE-RS	0.088	[0.059, 0.117]	5.96	<0.001
ROCKALL vs. HOLD-B	−0.110	[−0.175, −0.046]	−3.36	<0.001
ROCKALL vs. LACTATE/HB	−0.036	[−0.105, 0.032]	−1.03	0.301
GBS vs. AIMS65	0.156	[0.091, 0.222]	4.66	<0.001
GBS vs. H3B2	0.050	[0.012, 0.088]	2.55	0.011
GBS vs. ABC	0.107	[0.048, 0.166]	3.58	<0.001
GBS vs. ABL	0.017	[−0.034, 0.056]	0.48	0.628
GBS vs. PRE-RS	0.134	[0.076, 0.192]	4.51	<0.001
GBS vs. HOLD-B	−0.065	[−0.113, −0.017]	−2.65	0.008
GBS vs. LACTATE/HB	0.010	[−0.047, 0.066]	0.33	0.743
AIMS65 vs. H3B2	−0.107	[−0.173, −0.040]	−3.15	0.002
AIMS65 vs. ABC	−0.049	[−0.110, 0.012]	−1.58	0.114
AIMS65 vs. ABL	−0.140	[−0.211, −0.073]	−4.04	<0.001
AIMS65 vs. PRE-RS	−0.023	[−0.080, 0.035]	−0.78	0.437
AIMS65 vs. HOLD-B	−0.221	[−0.285, −0.158]	−6.80	<0.001
AIMS65 vs. LACTATE/HB	−0.147	[−0.218, −0.076]	−4.06	<0.001
H3B2 vs. ABC	0.057	[−0.007, 0.122]	1.74	0.082
H3B2 vs. ABL	−0.033	[−0.094, 0.020]	−1.27	0.205
H3B2 vs. PRE-RS	0.084	[0.026, 0.141]	2.86	0.004
H3B2 vs. HOLD-B	−0.115	[−0.167, −0.062]	−4.27	<0.001
H3B2 vs. LACTATE/HB	−0.040	[−0.100, 0.019]	−1.32	0.185
ABC vs. ABL	−0.091	[−0.163, −0.029]	−2.79	0.005
ABC vs. PRE-RS	0.026	[−0.021, 0.074]	1.09	0.275
ABC vs. HOLD-B	−0.172	[−0.234, −0.110]	−5.46	<0.001
ABC vs. LACTATE/HB	−0.098	[−0.168, −0.028]	−2.74	0.006
ABL vs. PRE-RS	0.117	[0.050, 0.189]	3.36	<0.001
ABL vs. HOLD-B	−0.081	[−0.123, −0.028]	−3.10	0.002
ABL vs. LACTATE/HB	−0.007	[−0.064, 0.063]	−0.01	0.991
PRE-RS vs. HOLD-B	−0.198	[−0.262, −0.135]	−6.13	<0.001
PRE-RS vs. LACTATE/HB	−0.124	[−0.193, −0.055]	−3.53	<0.001
HOLD-B vs. LACTATE/HB	0.074	[0.031, 0.118]	3.34	<0.001

**Figure A1:**
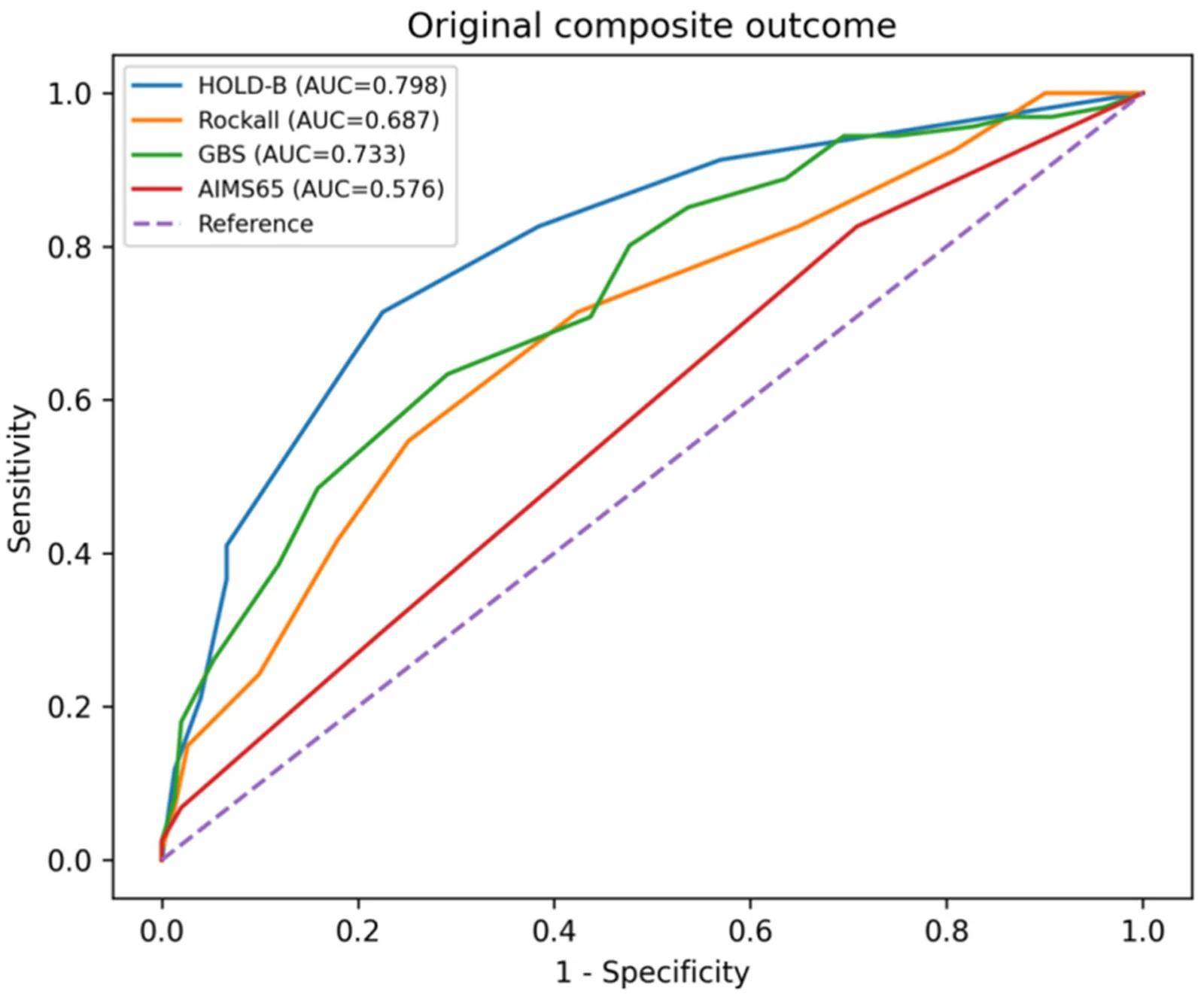
ROC Curves for Original composite endpoint.

**Figure A2:**
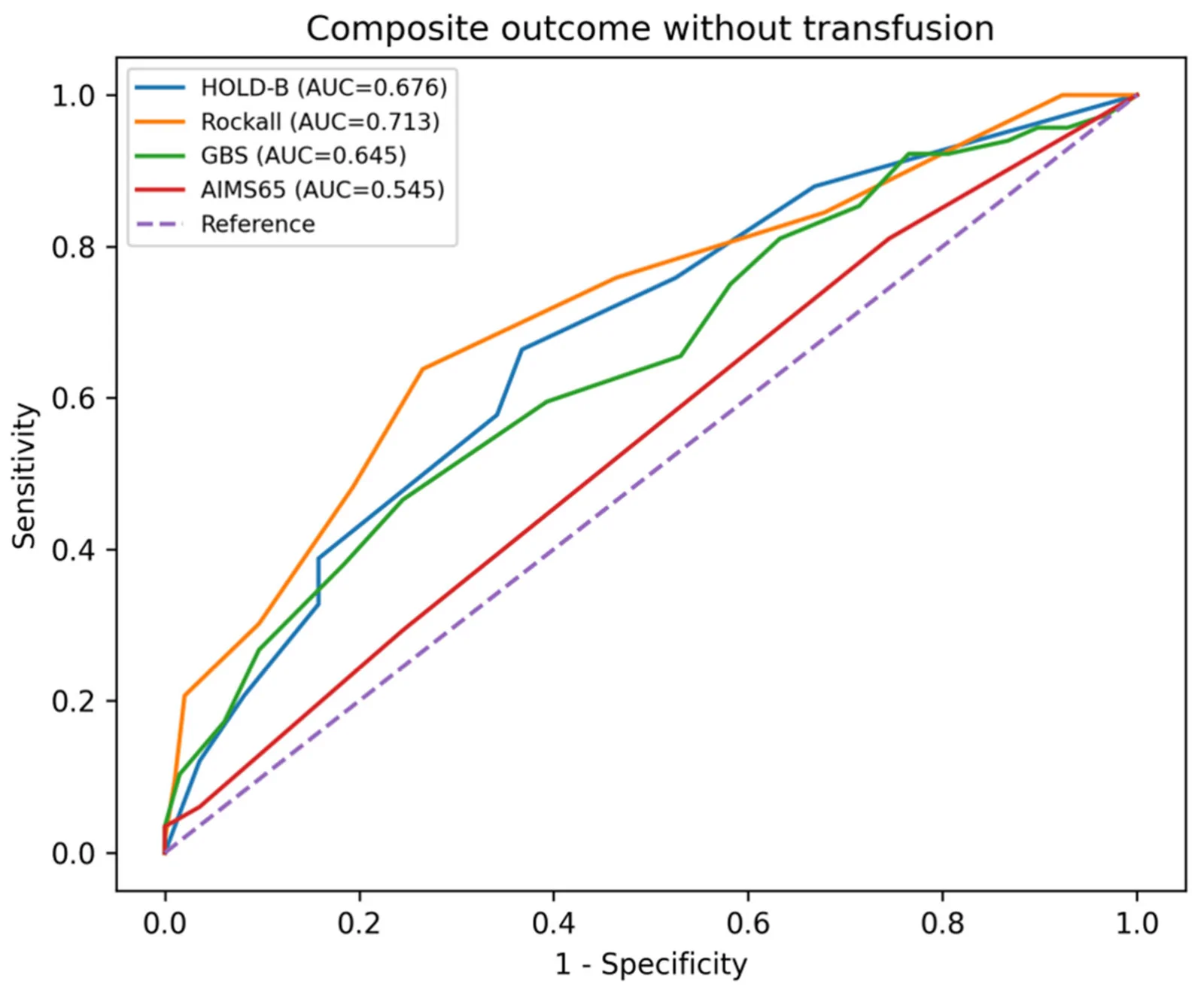
ROC Curves for Transfusion-Removed Composite Endpoints.

## Data Availability

Anonymized data supporting this study’s findings are available from the corresponding author upon reasonable request.

## Abbreviations

The following abbreviations are used in this manuscript:

ABL	Albumin, Blood urea nitrogen, Lactate score
ABC	Age, Blood tests, Comorbidities score
AIMS65	Albumin, INR, Mental status, Systolic blood pressure, Age ≥65 score
ALT	Alanine aminotransferase
AST	Aspartate aminotransferase
BUN	Blood urea nitrogen
CRP	C-reactive protein
DBP	Diastolic blood pressure
ED	Emergency department
GBS	Glasgow-Blatchford score
HOLD-B	Hemoglobin, Lactate, Diastolic blood pressure, Blood urea nitrogen
ICU	Intensive care unit
INR	International normalized ratio
PLT	Platelet
Pre-RS	Pre-endoscopic Rockall score
SBP	Systolic blood pressure
UGIB	Upper gastrointestinal bleeding
WBC	White blood cell
aPTT	Activated partial thromboplastin time

## Appendix A

### Appendix A.1

Percentages are calculated based on the total number of patients, which is 312.

**Table A1:** Distribution of Endpoint Components.

Component	n	%
Total patients	312	100.0
Original composite is high risk	161	51.6
Transfusion removed, high risk	116	37.2
Transfusion	88	28.2
Endoscopic procedure	79	25.3
Intensive care requirement	75	24.0
Rebleeding	9	2.9
Mortality	18	5.8

### Appendix A.2

Cut-off points are determined using the Youden index.

**Table A2:** ROC Analysis for Original Composite Endpoint.

Score	AUC [95% CI]	Cut-Off	Sensitivity	Specificity	PPV	NPV	Accuracy (ACC)
HOLD-B	0.798 [0.749–0.846]	≥4	0.714	0.775	0.772	0.718	0.744
Rockall	0.687 [0.629–0.745]	≥6	0.547	0.748	0.698	0.608	0.644
GBS	0.733 [0.678–0.788]	≥11	0.634	0.709	0.699	0.645	0.670
AIMS65	0.576 [0.518–0.635]	≥1	0.826	0.291	0.554	0.611	0.567
H3B2	0.683 [0.627–0.739]	≥2	0.739	0.517	0.620	0.650	0.631
ABC	0.626 [0.564–0.687]	≥4	0.571	0.636	0.626	0.582	0.603
ABL	0.716 [0.659–0.774]	≥5	0.500	0.856	0.786	0.619	0.673
Pre-RS	0.599 [0.538–0.661]	≥4	0.578	0.576	0.592	0.561	0.577

### Appendix A.3

In this analysis, high-risk endpoints have been redefined as endoscopic intervention, intensive care, rebleeding, or mortality.

**Table A3:** ROC Analysis for Transfusion-Removed Composite Endpoints.

Score	AUC [95% CI]	Cut-Off	Sensitivity	Specificity	PPV	NPV	Accuracy (ACC)
HOLD-B	0.676 [0.618–0.734]	≥4	0.664	0.633	0.517	0.761	0.644
Rockall	0.713 [0.652–0.773]	≥6	0.638	0.735	0.587	0.774	0.699
GBS	0.645 [0.580–0.703]	≥12	0.466	0.755	0.529	0.705	0.647
AIMS65	0.545 [0.481–0.598]	≥1	0.810	0.255	0.392	0.694	0.462
H3B2	0.645 [0.579–0.703]	≥2	0.750	0.464	0.453	0.758	0.571
ABC	0.580 [0.518–0.649]	≥4	0.560	0.582	0.442	0.691	0.574
ABL	0.561 [0.487–0.640]	≥2	0.827	0.295	0.404	0.747	0.490
Pre-RS	0.568 [0.506–0.631]	≥4	0.569	0.536	0.420	0.677	0.548

### Appendix A.4

*p*-values were calculated based on a paired DeLong AUC difference distribution. The positive difference favors HOLD-B.

**Table A4:** Differences in AUC Between HOLD-B and Other Scores in Original Composite Endpoint.

Comparison	AUC Difference [95% CI]	*p*
HOLD-B vs. Rockall	0.110 [0.049–0.173]	<0.001
HOLD-B vs. GBS	0.065 [0.019–0.113]	0.006
HOLD-B vs. AIMS65	0.221 [0.160–0.287]	<0.001
HOLD-B vs. H3B2	0.115 [0.065–0.165]	<0.001
HOLD-B vs. ABC	0.172 [0.105–0.233]	<0.001
HOLD-B vs. ABL	0.075 [0.027–0.126]	0.006
HOLD-B vs. pre-RS	0.198 [0.137–0.263]	<0.001

### Appendix A.5

*p*-values were calculated based on a paired bootstrap AUC difference distribution. The positive difference favors HOLD-B.

**Table A5:** Differences in AUC Between HOLD-B and Other Scores in Transfusion-Excluded Composite Endpoint.

Comparison	AUC Difference [95% CI]	*p*
HOLD-B vs. Rockall	−0.037 [−0.110–0.028]	0.254
HOLD-B vs. GBS	0.031 [−0.024–0.080]	0.270
HOLD-B vs. AIMS65	0.130 [0.060–0.197]	<0.001
HOLD-B vs. H3B2	0.031 [−0.027–0.085]	0.284
HOLD-B vs. ABC	0.095 [0.029–0.162]	0.008
HOLD-B vs. ABL	0.105 [0.057–0.157]	<0.001
HOLD-B vs. Pre-RS	0.108 [0.035–0.173]	0.002

### Appendix A.6

Predictors were included in the model based on the categorical threshold values of the HOLD-B components.

**Table A6:** Multivariate Logistic Regression for the Original Composite Endpoint.

Predictor	OR [95% CI]	*p*
Hemoglobin ≤ 8.05 g/dL	4.191 [2.420–7.259]	<0.001
Lactate > 2.15 mmol/L	2.751 [1.535–4.930]	<0.001
Diastolic BP ≤ 67.5 mmHg	1.992 [1.173–3.384]	0.011
BUN > 44.5 mg/dL	3.390 [1.858–6.187]	<0.001

### Appendix A.7

When transfusion was excluded from the composite endpoint, hemoglobin was no longer an independent predictor.

**Table A7:** Multivariate Logistic Regression for Transfusion-Excluded Composite Endpoint.

Predictor	OR [95% CI]	*p*
Hemoglobin ≤ 8.05 g/dL	0.848 [0.500–1.440]	0.542
Lactate > 2.15 mmol/L	3.392 [2.013–5.714]	<0.001
Diastolic BP ≤ 67.5 mmHg	1.504 [0.896–2.524]	0.122
BUN > 44.5 mg/dL	2.279 [1.326–3.917]	0.003

### Appendix A.8

The original model demonstrated acceptable calibration. In the transfusion-excluded model, a statistically significant Hosmer–Lemeshow test suggested reduced calibration performance.

**Table A8:** Bootstrap Internal Validation and Calibration Summary.

Model	Apparent AUC	Optimism-Corrected AUC	Average Optimism	Hosmer–Lemeshow χ^2^	df	*p*
Original composite	0.802	0.796	0.006	5.108	6	0.530
Transfusion-excluded composite	0.723	0.712	0.011	15.308	6	0.018
